# Needs & networks: understanding the role and impact of social networks on HIV (self-)testing among GBMSM and trans people in England and Wales

**DOI:** 10.1186/s12889-024-18487-w

**Published:** 2024-07-25

**Authors:** Isaac Yen-Hao Chu, Peter Weatherburn, Talen Wright, Phil Samba, Emily Jay Nicholls, Leanne McCabe, Mitzy Gafos, David T Dunn, Roy Trevelion, Fiona M Burns, Alison J Rodger, T Charles Witzel

**Affiliations:** 1 Institute for Global Health, University College London, Royal Free Hospital, Rowland Hill Street, NW3 2PF London, UK; 2 Faculty of Public Health and Policy, London School of Hygiene and Tropical Medicine, 15-17 Tavistock Place, WC1H 9SH London, UK; 3 Division of Psychiatry, University College London, 149 Tottenham Court Rd, W1T 7BN London, UK; 4 The Love Tank CIC, The Green House, 244-254 Cambridge Heath Road, E2 9DA London, UK; 5 Medical Research Council Clinical Trials Unit at University College London, 90 High Holborn, WC1V 6LJ London, UK; 6 HIV i-Base, 107 The Maltings, 169 Tower Bridge Road, SE1 3LJ London, UK

**Keywords:** HIV self-testing, Social network, Needs, MSM, Trans, United Kingdom

## Abstract

**Background:**

Understanding how HIV self-testing (HIVST) can meet the testing needs of gay, bisexual and other men who have sex with men (GBMSM) and trans people whose social networks vary is key to upscaling HIVST implementation. We aim to develop a contextual understanding of social networks and HIV testing needs among GBMSM (cis and transgender) and trans women in SELPHI (An HIV Self-testing Public Health Intervention), the UK’s largest randomised trial on HIVST.

**Methods:**

This study re-analysed qualitative interviews conducted from 2015 to 2020. Forty-three in-person interviews were thematically analysed using the Framework Method. Our analytic matrix inductively categorised participants based on the unmet needs for HIV testing and the extent of social network support. The role of social networks on HIVST behaviour was explored based on individuals’ testing trajectories.

**Results:**

Four distinct groups were identified based on their unmet testing needs and perceived support from social networks. Optimisation advocates (people with high unmet needs and with high network support, *n* = 17) strived to tackle their remaining barriers to HIV testing through timely support and empowerment from social networks. Privacy seekers (people with high unmet needs and with low network support, *n* = 6) prioritised privacy because of perceived stigma. Opportunistic adopters (people with low unmet needs and with high network support, *n* = 16) appreciated social network support and acknowledged socially privileged lives. Resilient testers (people with low unmet needs and with low network support, *n* = 4) might hold potentially disproportionate confidence in managing HIV risks without sustainable coping strategies for potential seroconversion. Supportive social networks can facilitate users’ uptake of HIVST by: (1) increasing awareness and positive attitudes towards HIVST, (2) facilitating users’ initiation into HIVST with timely support and (3) affording participants an inclusive space to share and discuss testing strategies.

**Conclusions:**

Our proposed categorisation may facilitate the development of differentiated person-centred HIVST programmes. HIVST implementers should carefully consider individuals’ unmet testing needs and perceived levels of social support, and design context-specific HIVST strategies that link people lacking supportive social networks to comprehensive HIV care.

## Background

Since 2016, HIV incidence among gay, bisexual and other men who have sex with men (GBMSM) living in the UK has declined rapidly with recorded increases in the uptake of testing, treatment and prevention measures (including pre-exposure prophylaxis (PrEP)) for HIV [1]. Nevertheless, such success is not equitable. GBMSM and trans people are highly heterogenous with interconnected lived experiences of marginalisation related to their gender identity, sexual orientation, ethnicity and perceived social connectedness. Recent UK studies have documented high unmet needs in these populations and the barriers they encounter to utilising sexual health services, including perceived HIV stigma in clinical settings, limited availability of facility-based HIV testing and inaccessible HIV PrEP [2–4]. Such unmet needs are also distributed unevenly at various timepoints over the life-course of GBMSM and trans individuals, whose life-course involves ever-changing interactions with self-identity, interpersonal relationships and social norms [5, 6].

Research arising from SELPHI (An HIV Self-Testing Public Health Intervention) has generated a substantial body of evidence on HIV self-testing (HIVST), defined as approaches where an individual uses a rapid diagnostic test for HIV and interprets their own results. Remaining the largest HIVST study in high-income settings, SELPHI was an innovative online randomised controlled trial that allocated free blood-based HIVST kits to 10,111 GBMSM (both cisgender and transgender men) and 24 trans women living in England and Wales [7]. With a high self-reported testing uptake (95%, 4263/4511) among those receiving free HIVST kits [7], SELPHI has demonstrated the feasibility and acceptability of HIVST among GBMSM and trans women without reducing linkage to sexual health care in the UK. Formative research [8, 9] that developed interventions used in SELPHI highlighted the potential for multi-level barriers to using HIVST, such as fear of seroconversion and a perceived lack of testing support. Furthermore, there remain unanswered questions about how broader social contexts and interpersonal relationships may influence the uptake of HIVST based on individual differences in both HIV testing need and social networks.

Implementation science researchers have underlined the pertinence of social networks in facilitating HIV testing behaviours as well as the role of social support in improving personal well-being [10–12]. Many studies have quantitatively investigated the number of networks, the strength of network ties and statistical probabilities in knowledge translation and behaviour change [13–15]. In line with World Health Organization recommendations on utilising social networks to promote HIV testing [16], several implementation studies have highlighted how social networks can be harnessed to increase the uptake of HIV testing among GBMSM and trans people [17–19]. In the UK, we have demonstrated that weak or unsupportive social networks may exacerbate unmet health needs of GBMSM and trans people [2]. Witzel et al. also reported that some GBMSM in SELPHI developed testing patterns to conform to expectations from other men, public health authorities and social norms, highlighting the influence of peers in HIV testing [9].

Following SELPHI’s preliminary explorations of social networks, key questions remained as to how HIVST can meet the needs of GBMSM and trans women whose social networks vary substantially. There is a need to better understand how social networks impact individuals’ uptake of HIVST. Such understandings can shape HIVST roll-out by informing strategies that account for the role of social networks in a landscape of diverse HIV testing services (HTS) provision, including facility-based testing, community testing initiatives and HIV self-sampling [20]. This qualitative study aims to develop contextual understandings of the interplay between social networks and HIV testing need among GBMSM and trans women in SELPHI.

## Methods

### Study design

This study re-analysed qualitative datasets from SELPHI. The protocol and previous outcomes of SELPHI have been published elsewhere [7, 21–24, 25]. As SELPHI generated a large amount of qualitative data throughout its formative phase, trial period and subsequent studies, we were aware of emerging narratives about social networks and thus initiated this study (All but IYC were involved in the data collection of SELPHI and its sub-studies). We applied secondary data analysis to understand how support from social networks influenced attitudes and decision-making towards HIVST among individuals whose testing needs varied.

### The SELPHI qualitative dataset

The dataset comprised transcripts of six focus group discussions in the pre-trial formative phase (with 47 GBMSM recruited via mobile apps and social media) and 86 semi-structured individual interviews with 66 cis-gender GBMSM and 20 trans people who were sampled purposively from SELPHI trial participants volunteering for follow-on interviews. All interviews and group discussion were in English. Interviews were conducted by EJN, PS, TCW and TW either online or in-person, whereas focus groups were facilitated by TCW and PW in-person. Interviews were audio recorded, transcribed verbatim, anonymised and delinked from SELPHI trial data. Table 1 summarises the study period, study aim, participant numbers in the pre-trial formative phase and three sub-studies constituting the total qualitative dataset. The details of participant recruitment and data collection of each study are reported elsewhere [2, 8, 24, 26].

**Table 1:** An overview of the SELPHI qualitative dataset

Study name	Time of data collection	Number of participants	Number of interviews included in the analysis	Aim of the study	Reference for full study descriptions
Formative phase	2015 (Prior to SELPHI)	Six focus groups (Five to nine people per group) with 47 cis-gender MSM	Not applicable	Explore HIVST motivation, values and preferences among MSM communities	Witzel et al., 2016 [8] Witzel et al., 2017 [9]
Qualitative sub-study	May 2017– Oct 2018	37 interviews with cis-gender MSM	10	Understand the acceptability and function of HIVST intervention in SELPHI RCT	Witzel et al., 2020 [26] Witzel et al., 2020 [27]
Trans sub-study	Apr 2019– Oct 2019	20 interviews with trans people	10	Explore HIVST experiences among trans people in the SELPHI RCT	Witzel et al., 2021 [24] Wright et al., 2021 [28]
Asian, Black and Latin American (ABLA) men sub-study	Apr 2020– Jul 2020	29 interviews with cis-gender MSM self-identifying as Asian, African, Caribbean, Latin American or mixed ethnicity	23	Explore the experiences of ABLA men in the SELPHI RCT	Nicholls et al., 2022 [2]

### Data analysis

We employed the Framework Method [29] for data analysis to enable our cross-disciplinary team to systematically analyse and compare qualitative data collected across three sub-studies. We did not present data from the pre-trial formative phase (see elsewhere [8, 9] for details) as they were only utilised to enhance trustworthiness in our analytical framework. Informed by testing trajectory and the HIVST mechanism of action proposed by Witzel et al. [27], our framework explored the relationship between SELPHI participants’ social networks and HIVST behaviours (i.e., before, during and after using an HIVST kit).

After IYC reviewed the literature on HIV testing regarding the relationships between individuals and social networks, we decided to focus on the interplay between two domains: the level of support gained from social networks and the extent of unmet needs for testing. Firstly, a network was considered *supportive* if participants felt confident in talking about HIV and HIV testing within it. If individuals either (1) had family or friends who stigmatised sexual minorities and/or HIV or (2) were unwilling to disclose their needs for HIV testing and sexual health services within their networks, they were considered to be in an *unsupportive* network. Secondly, we defined individuals with *high unmet need* as those who perceived themselves to have unmet HIV testing needs or felt that HTS in the UK failed to meet their specific needs. Individuals who perceived no need for HIV testing or whose needs were satisfied by existing HTS were defined as having *low unmet need*.

Our analysis comprised three iterative steps. Firstly, IYC familiarised himself with the dataset by reading all transcripts twice. IYC then developed an analytical framework on needs and networks, discussing and revising it with TCW, PW and the patient and public involvement group (comprising GBMSM and trans women in England and Wales who did not join SELPHI but provide person-centred feedback on data analysis, data interpretation and dissemination of study results). As none of the sub-studies were originally designed for our specific research inquiries under time constraints, we decided to sample half (43 of 86) of the interview transcripts for data analysis. We mainly focussed on the sub-study of Asian, Black and Latin American (ABLA) men because its topic guide contained questions on social network size and configuration. Specifically, IYC initially included 23 transcripts from the ABLA sub-study [2]. He then randomly included 10 transcripts from the Qualitative sub-study and 10 transcripts from the Trans sub-study [24–27].

Secondly, IYC applied the matrix to two interview transcripts alongside TCW, compared codes and reached consensus on the operational definitions of emerging themes and typologies. Both IYC and TCW are cis-gender men with years of experience in conducting qualitative data analyses using decolonised and phenomenological approaches. Codes included unmet needs (high/low), social network (supportive/unsupportive), experiences in HIV testing, roles of HIVST in HTS and coping strategies. IYC then identified more codes that were relevant to ‘support’ and ‘HIV testing needs’ in each transcript with agreed operational definitions in a codebook file. The needs-network matrix was further refined through IYC’s engagement with the broader SELPHI dataset. IYC continued indexing and charting codes, retaining diversity and balance in data analysis by including all sampled transcripts until conceptual clarity was reached without newly emerging themes.

Finally, after IYC analysed 43 interview transcripts, the research team reviewed the findings to reach consensus and to ensure that the analytical matrix, thematic interpretation and selected quotes were logical and coherent. QSR NVivo 12 was utilised for data organisation.

### Ethical considerations

 Ethical approval of this secondary data analysis was granted by University College London Research Ethics Committee (Ref: 24477.001).

## Results

The 43 SELPHI participants included in this analysis were diverse in terms of HIV testing histories, gender identity, sexual orientation and ethnic background (Table 2). The majority of participants were cis-gender GBMSM, ethnic minorities and had medium to high levels of education. Most (38 of 43) interviewees received and used HIVST kits throughout SELPHI.

**Table 2:** Demographics of the 43 analysed interviewees

Demographic	Category	Count (n = 43)
Age	18–25	13
	26–35	12
	36–45	8
	46+	10
Gender	Cis man	33
	Trans man	5
	Trans woman	5
Sexual orientation	Bisexual	5
	Heterosexual/Straight	2
	Homosexual/Gay	31
	Others/undisclosed	5
Ethnicity	Asian	5
	Black	4
	Latin American	6
	Mixed	14
	White	14
Higher education qualifications	Low*	6
	Medium**	15
	High***	22
HIV testing history at SELPHI trial enrolment	Never tested	6
	Less than three months	6
	Three to six months	10
	Seven to 12 months	10
	More than 12 months	11
Received HIV self-testing kits (one or more)	Yes	38
	No	5

* General Certificate of Secondary Education (leaving official education at age 16) and below

** A-levels or equivalent higher education qualifications

*** Degree(s) or higher

SELPHI: An HIV Self-testing Public Health Intervention

Informed by our thematic focus on needs and networks, we present four groups with distinct perspectives on HIVST using a two-by-two need-network matrix. Our analysis revealed that individuals’ response to HIVST were determined by their unmet needs for HIV testing and associated support gained from social networks. To facilitate contextual explorations (i.e., GBMSM and trans women having various ethnic background and HIV testing histories) of individual perspectives, we firstly described distinctive features and norms of each group. We then highlight what roles supportive social networks can play to maximise the benefits of HIV self-testing for GBMSM and trans women in England and Wales.

Our analysis demonstrated the ways that GBMSM and trans people understand HIV testing and how their testing behaviour varies based on the interaction of two key factors: [1] individual’s unmet needs for HIV testing and [2] the extent to which an individual has supportive social networks. Overall, supportive social networks facilitated the development of positive HIV testing norms for GBMSM and trans people, strengthened their capabilities to meet their needs for HIV testing and shaped their views on the acceptability of HIVST. Those without supportive social networks relied on their own capabilities to manage perceived risks of HIV acquisition, access HIV testing services (HTS) and cope with anxiety about potential seroconversion in the context of HIV stigma.

### A typology of distinct perspectives on HIVST

**Fig. 1:**
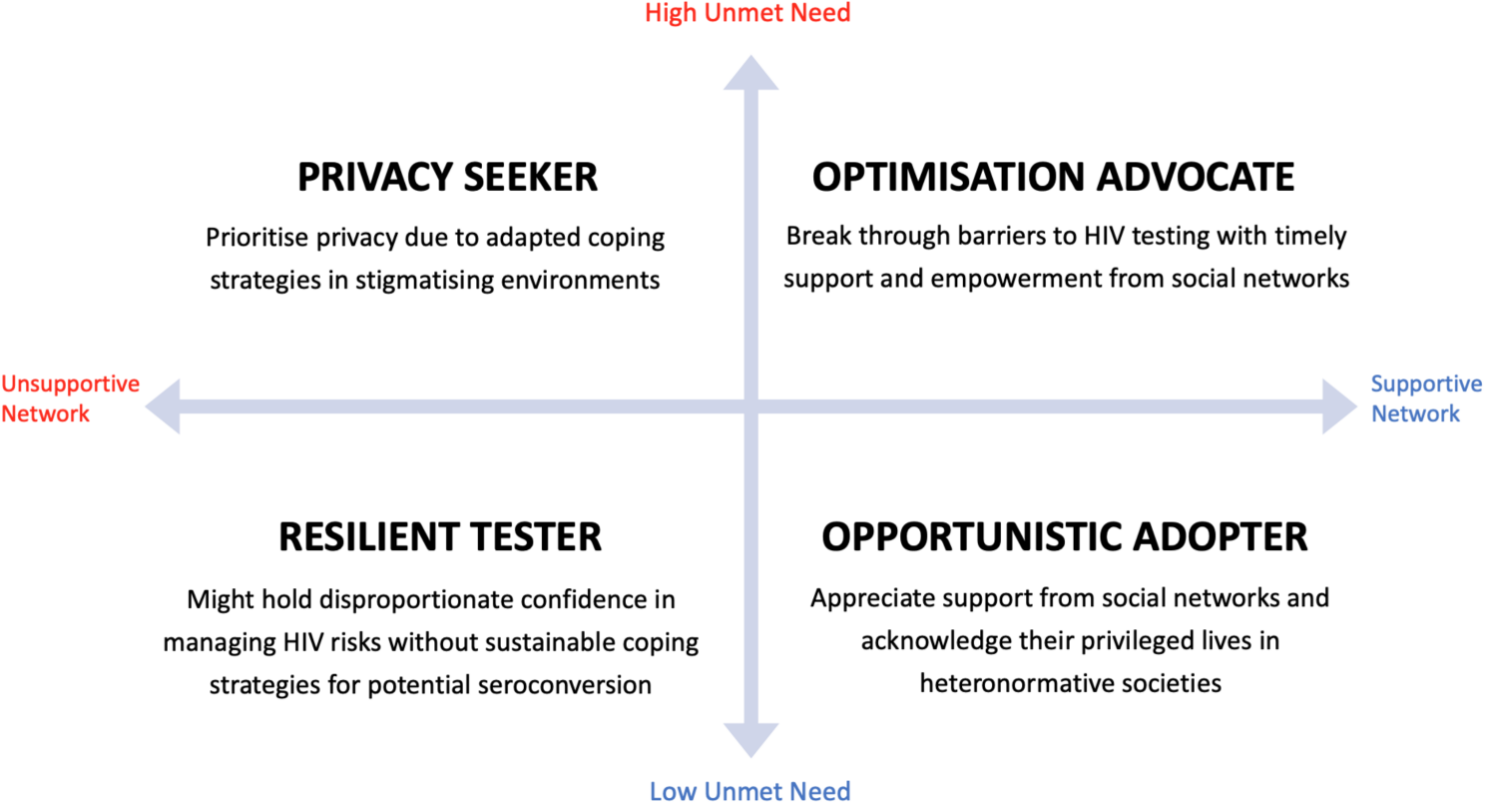
Features of four types of HIVST users by unmet testing need and social network support

Using our matrix of testing needs and social networks, we identified four groups based on their distinct perspectives on HIV self-testing: optimisation advocates (*n* = 17), opportunistic adopters (*n* = 16), privacy seekers (*n* = 6) and resilient testers (*n* = 4). Figure 1 summarises the defining features and key descriptions of the four groups. In the following, we present key features of, and perceived social norms in, each group.

#### Optimisation advocates

Individuals in this group reported a wide range of impediments to accessing HTS, but they managed to test for HIV nonetheless. All highlighted barriers to utilising HTS in England and Wales, such as geography, limited clinical opening hours, long waiting time at clinics and inconvenient timeslots for appointments. Many individuals advocated the use of HIVST as an optimal testing strategy for themselves as it greatly improved their overall HIV testing experience. Almost all were sexually active, had tested for HIV before joining SELPHI (except for one with a failed attempt to test for HIV), and had social networks from diverse backgrounds regarding gender and sexuality.

Optimisation advocates often discussed their sexual practices, the stigma of HIV and challenges in accessing HTS with supportive social networks (e.g., friends, family members and partners). They described how support from their social networks facilitated access to available testing opportunities in HTS. For example, members of their social networks offered support by acknowledging their challenges, encouraging them to engage with HTS and providing information on non-facility-based testing strategies (including HIVST). Such support further established positive norms for regular HIV testing in this group, so optimisation advocates could manage their ongoing needs for HIV testing.

If someone had a bit of [HIV] scare or anything, definitely we are there to have each other’s back and, literally and figuratively, hold each other’s hand through it and just make sure that everyone’s okay… I think it’s quite important just to have that support group and people around you that understand what you’re going through and understand the process [of HIV testing] and understand what that means or what’s happening. That, for me, is quite important.

(20-year-old Black gay man, tested in the last 12 months, optimisation advocate)

I usually test [for HIV] every month if I am sexually active or not in a relationship with a trusting partner. Luckily my friends that are both gay and straight are supportive, and we talk openly about sex. Therefore, nothing is off topic; nothing is too bad to talk about.

(21-year-old gay man with a mixed ethnic background, self-tested, optimisation advocate)

#### Opportunistic adopters

Living in supportive social networks, all individuals in this group felt satisfied with existing HTS. While most were aware of negative norms surrounding HIV and sexual minorities in society, they described how supportive networks boosted their confidence in utilising HTS routinely. They also held strong, positive norms around HIV risk management and HIV testing, linking both to bio-citizenship as being ‘good’ GBMSM and trans women. Opportunistic adopters were more likely to use HIVST for satisfying testing need if unable to access HTS, or to respond to positive norms around frequent testing. They tended to consider HIVST as an innovative alternative to existing services. Such thoughts were particularly common in interviews following the onset of the COVID-19 pandemic, which reduced access to HTS and helped normalise self-testing.

Most opportunistic adopters highly valued the support from social networks. They acknowledged their relatively ‘privileged life’ (compared to others without support) in heteronormative societies where the stigma surrounding sexual/gender minorities, people living with HIV and sexual health service users were pervasive. This group tended to describe having their needs well met by existing HTS. They felt competent in managing their HIV risk and seeking support from members of their networks if needed.

I think definitely because of local LGBT community and events, I’ve been very active, and I went to like an LGBT youth group from when I was like 14 [years old] onwards. And then when I went to university, I was part of the LGBT society and ran it at one point…. And all of them have just had a lot of really great activists and positive encouragement around HIV testing.

(20-year-old White bisexual trans man, tested in the last six months, opportunistic adopter)

I know I have had a very easy, privileged life. And I know that living in London, it’s a bubble. So [getting HIV tests has] never been an issue. And it’s always been very easy. But I know I’m very privileged and have a very easy life.

(49-year-old gay man with a mixed ethnic background, self-tested, opportunistic adopter)

#### Privacy seekers

Embedded in networks they perceived to be unsupportive, this group were sexually active and felt that the current HTS did not satisfy their testing needs. Privacy seekers prioritised privacy in HIV testing as they had developed coping strategies to live in environments that stigmatised HIV, HIV testing and sexual/gender minorities. Some had experienced discriminatory behaviour in existing HTS, whereas others felt unable to disclose their sexual orientation or gender identity due to conservative living environments.

I told my mother once that I was having [an] HIV test about ten years ago [as] I literally thought it was a good thing. She freaked out… she was like: ‘Why are you having a test? Why do you need a test? I have never had a test and I am 50 [years old]!’…. So I thought, actually, it [HIV testing] is not as widespread as I thought. So, I just stopped talking about it [HIV testing], really.

(35-year-old Black bisexual man, self-tested, privacy seeker)

Most privacy seekers experienced pervasive negative stigmas surrounding gender identity (e.g., transphobia), sexual orientation (e.g., homophobia) and HIV from their environments or family values. They considered HIVST to be a gamechanger that enabled them to meet their testing needs without any disclosure of sexual or gender identity. The private and confidential nature of HIVST was a crucial facilitator for privacy seekers, affording them reassurance of their HIV status without risking disclosure in HIV testing services. However, when their HIVST results showed positive, some reported feeling desperate, alone and vulnerable in seeking confirmatory testing and HIV care.

Interviewer: So how, if at all, does that [HIV-positive results] overlay or interact with other aspects of your identity?

Participant: It does hugely because I’m not out. So then the friend that I told.… I told her that I had [HIV], and then she knew I was gay. But it was very much… it feels like, oh, ‘I’m gay and I’ve got HIV’. It’s like, oh no, shit, double whammy, in some ways. And that’s my biggest fear about telling my family because it’s a… I’m going to label it as two disappointments because that’s how it feels.

(36-year-old gay man with a mixed ethnic background, tested in the last six months, privacy seeker)

#### Resilient testers

Having low unmet needs and living in unsupportive networks, participants in this group demonstrated resilience in managing their needs for HIV testing and tended to be confident in managing their risk of HIV acquisition. Some were perhaps disproportionately confident in their management of HIV risk, so they rarely tested for HIV due to limited perceived need. Others accessed facility-based and self-sampling testing services to satisfy their needs. For those who utilised existing services, it was not clear whether they had robust coping strategies for potential seroconversion.

Interviewer: Did you think there was a possibility of a positive result [for HIV]?

Participant: I didn’t think so because there wasn’t any kind of activity in my mind that I partake in that would have resulted in that [HIV acquisition]. But even if there was, it wouldn’t been something in my mind anyway to say this [being HIV-positive] might happen. Because I’ll be thinking about things that might happen [if I am HIV-positive], and that’s not going to do me any good.

(18-year-old Asian gay man, self-tested, resilient tester)

When worrying about seroconversion, one interviewee could not seek support from his social networks but instead did so from HTS, which often did not immediately respond to their emotional needs.

I have always gotten them [HIV testing] done in some sort of public clinics […], so basically, during the session, they [staff at clinics] asked about my [sexual] history and then, you know, basically reminded me to wear condoms, etc. But there was no kind of major counselling and no kind of post-session follow-up.

(31-year-old gay man with a mixed ethnic background, self-tested, resilient tester)

### Roles of social networks on HIVST uptake

To better understand the potential roles of social networks on participant’s uptake of HIVST, we identified three temporal phases of an individual’s journey of HIV self-testing: before, during and after using the HIVST kit. Under each theme, we compared perspectives across the four distinct groups (i.e., optimisation advocates, opportunistic adopters, privacy seekers and resilient testers) to demonstrate how HIVST uptake may vary by their social network support and unmet testing needs.

### Before HIVST: awareness, attitude and experience

Supportive social networks made GBMSM and trans people more aware of HIVST, and more likely to hold positive attitudes towards HIVST based on their previous testing experiences. While most interviewees reported that their primary sources of information on HIVST were SELPHI’s advertisements on social media and geosocial applications, several optimisation advocates and opportunistic adopters highlighted social networks’ effects on their positive attitudes to HIVST. Members in their supportive networks (e.g., friends, family and peers in LGBT + community groups) often disseminated information on accessible HIV testing channels (e.g., HIVST and the SELPHI trial), reminded participants of the importance of HIV testing and encouraged them to establish and maintain testing routines. For example, a trans woman recalled how a friend shared information on HIVST and encouraged her to join the SELPHI trial.

I remember when I saw it [SELPHI] because it was [from] a friend of mine. He told me about that. Because he… I think he joined the [SELPHI] study as well. And he thought that I wanted as well to participate…. [because] they send you the test-at-home [kits]. I was [like], ‘What? At home? That’s amazing! I want to try it!’ Yes. Because [testing HIV] at home it’s very easy.

(42-year-old Latin American trans woman, self-tested, opportunistic adopter)

Paradoxically, unsupportive social networks may also foster awareness of HIVST among some GBMSM and trans women through other mechanisms. None of the participants living in unsupportive networks (i.e., privacy seekers and resilient testers) learnt about HIVST from their social networks. Nevertheless, some argued that, because they were unable to disclose their testing needs in stigmatising social networks and living environments, they sought every opportunity to get information on HIVST and other non-facility-based testing channels. One resilient tester noted how he found SELPHI online when worrying about HIV acquisition after condomless sex.

I was afraid and scared [of getting HIV], so I didn’t know what to do. And I was very ashamed to go to the hospital. So, I tried to see if there was any self-test to do at home. So, then I found you [SELPHI] and I was like, ‘okay, I’m going to try it’. And I tried and it was pretty nice, and it was the next two-year relationship doing the programme.

(24-year-old Latin American gay man, self-tested, resilient tester)

Hence, due to the absence of supportive social networks, privacy seekers and resilient testers were more likely to access information from webpages compared to optimisation advocates and opportunistic adopters. Those equipped with digital and English literacy were more likely to access information on HIVST online.

It is worth noting that those with higher unmet needs may express greater interest in using HIVST. For example, compared with resilient testers whose needs were satisfied by current services, most privacy seekers expressed enthusiasm towards HIVST by highlighting its convenience and efficiency.

I think it [HIVST] is brilliant! Unbelievable! The fact that you can get that for something that had been a very arduous process, and the fact that you can actually get that done in your home. I was like, if you could buy them off the shelf, I’d keep five or six of them at home…. I loved the idea. I thought it was an absolute gamechanger.

(48-year-old gay man with a mixed ethnic background, self-tested, privacy seeker)

### During HIVST: motivation, action and support

Our analysis did not identify specific patterns in how social networks motivated participants to adopt HIVST. Regarding motivation for joining SELPHI, some people with supportive networks expressed altruistic reasons, such as helping GBMSM and trans communities by contributing to scientific advancement. Overall, reasons for engaging in HIVST included worries about HIV, wanting reassurance about HIV-negative status and curiosity about innovative technologies that complemented, or potentially substituted, existing HTS.

We found, however, that supportive social networks played key roles in facilitating users’ initiation into HIVST by offering timely and personal support throughout the process of self-testing. Some participants recognised that such networks helped them tackle challenges at each step of HIVST, such as setting up the HIVST kits, following instructions and overcoming their fear of obtaining a fingerpick blood sample. When awaiting their self-testing results, several optimisation advocates acknowledged the support from their friends or family who either stayed nearby or supported them in interpreting testing results.

I actually did it [HIVST] [at] work. Because the person who told me about it, I work with him and we’re only a small team. So, I think it was only the two of us in the office. So, it [HIVST kit] got delivered to the office because all my mail goes there because I’m not home. He did it [HIVST beforehand]. So, I was like, I’ll do mine…. He helped me with the kit and guided me along. But yes, because he’s my friend, I know that whatever happened, I’d be able to talk to him.

(25-year-old White gay man, self-tested, optimisation advocate)

Compared with optimisation advocates, those with low unmet needs (i.e., opportunistic adopters) tended to express more confidence in doing HIVST without requesting support from social networks. Conversely, privacy seekers and resilient testers received little or no psychosocial support during HIVST. Most were motivated by their perceived increased risk of HIV acquisition (e.g., having condomless sex or increased numbers of sexual partners). They coped alone with negative emotions (e.g., anxiety or fear of HIV-positive results) arising from HIV stigma and uncertainties in testing procedures at each step of HIVST. Particularly, when facing difficulties in operating self-testing kits or interpreting results, they often coped with their distress by relying on instructions provided with the HIVST kit due to a lack of interpersonal support. When unsure about the results of HIVST or constantly worrying about getting HIV, most preferred seeking reassurance and potential support from medical professionals at facility-based HTS.

Participant: I was kind of scared that I had HIV at the time. So, I went ahead [to a testing facility] and then done the tests…. I was given counselling [like] ‘if it does turn out to be positive, don’t be frightened. There is medication, there is this, there is that’. So, I was given some counselling before the [HIV] test”.

Interviewer: Were you given any support around when you got the result?

Participant: It was quite normal to be fair, I got the result, it was negative, so I just walked out. That’s about it.

(28-year-old Black gay man, self-tested, resilient tester)

### After HIVST: sharing, discussion and influence

Supportive social networks afforded GBMSM and trans people a safe space to share their experiences of HIVST and discuss future strategies for HIV testing. Most optimisation advocates contended that they felt empowered by such sharing with their social networks. As most participants did self-testing at venues where they interacted with social networks (e.g., their own home or other types of residences), non-judgemental conversations about the efficiency and convenience of HIVST provided participants with opportunities to further strengthen their ties with their family, partners and friends. Because HIVST was relatively innovative, optimisation advocates tended to become innovators in introducing HIVST to their networks.

I think it was surprising to people about how easy it [HIVST] was to do because obviously some people, they think it’s a… well, most people I spoke to thought you had to go for this big old blood test, and they go, and they have to go through this process of doing this way…. It [HIVST] is so easy; I think [that it] was a good idea for them.

(22-year-old White trans man, never self-tested, optimisation advocate)

Like optimisation advocates, opportunistic adopters shared their experience of HIVST with network members. However, most did not actively discuss HIVST with their social networks. Many opportunistic adopters felt satisfied with their current testing strategies or already had specific testing routines, so their needs for HIVST were limited.

Most privacy seekers and resilient testers did not talk about HIVST within their social networks. They did not feel safe initiating conversations about HIV and HIVST due to anticipated moral judgements and potential for inadvertent disclosure of their gender identities and sexual orientation. Some privacy seekers reported that they only shared the HIVST results with healthcare workers who practised outside their own residential areas to prevent the possibility of their social networks from knowing their HIV testing behaviour. Despite facing difficulties in conservative living environments, many acknowledged HIVST as a transformative and empowering innovation.

It [HIVST] felt empowering, actually. I mean that quite specifically. The fact that I can do something, and I can actually go and do the [HIV] test….

I have revised as What I really love about the SELPHI testing kit is that I can do it without being judged.

(48-year-old gay man with a mixed ethnic background, self-tested, privacy seeker)

## Discussion

Re-analysing qualitative data from 43 GBMSM and trans women in the SELPHI trial, our study identified four distinct groups (i.e., optimisation advocates, opportunistic adopters, privacy seekers and resilient testers) defined by their unmet needs for HIV testing and perceived support from social networks. We also revealed how social networks affected SELPHI participants before, during and after HIVST. Overall, supportive social networks may encourage optimisation advocates to adopt HIVST by affording them information before, holistic support during, and empowering spaces to share experiences after testing. Such networks also enable opportunistic adopters to consolidate their testing routines by utilising HIVST and existing HTS. Receiving little social support, most privacy seekers viewed HIVST as a gamechanger to satisfy their testing needs without any disclosure, whereas resilient testers often held strong confidence in self-managing their HIV risk. Both privacy seekers and resilient testers were prone to internalised stigmas surrounding gender identity, sexual orientation and testing for HIV. Such stigmas impact on their coping strategies for potential seroconversion and capacities to interact with social networks.

Our four categorisations present a plausible spectrum for researchers, health promotors and policymakers to understand the life-course [5, 6, 30] of GBMSM and trans women. One can imagine that, in heteronormative societies, most GBMSM and trans women started their HIV testing and sexual health journey as privacy seekers who had substantial testing needs without sufficient support from networks. After interacting with the social norms within their living environments, individuals receiving support from new networks may turn into optimisation advocates. Those who do not experience or receive network support may become resilient testers by relying on available HTS. Ultimately, in suitable circumstances, they may become opportunistic adopters when they meet testing needs, have supportive networks and routinely test for HIV to maintain well-being. It is worth noting that individuals may shift among four categorisations owing to potential changes in their testing needs (e.g., being more/less sexually active), accessibility of HTS (e.g., opening/closing times of testing facilities) or the configuration of their social network (e.g., moving to new environments or interruptions of relationships). Our needs-network typology has great potential in advancing the understanding of the life-course of HIV testing among sexual minorities in similar contexts, warranting further research.

Our findings indicated that social networks may not determine participants’ awareness and interests in using HIVST, which were inconsistent with the findings from Canada and China [17, 31, 32]. We propose two probable explanations. Firstly, as SELPHI participants were predominantly recruited from online platforms and mobile applications, they may under-report the roles of ‘offline’ social networks in raising awareness of HIVST. Secondly, it is feasible that social networks might not be pivotal to HIVST awareness, but to initiation of HIVST use. Some participants underlined that supportive networks were key influences on their HIVST behaviour by offering timely assistance and space to share and discuss HIVST, which may facilitate the formation of positive testing norms and routines.

To our knowledge, this is the first study demonstrating contextual understandings of GBMSM and trans people’s HIVST through the lens of unmet testing needs and social network support. Our findings extend Witzel et al.‘s explorations of GBMSM’s testing typology [27] by offering in-depth analyses of the self-testing journey among GBMSM and trans women. Our proposed user typology and identified roles of social networks not only advance knowledge of differentiated HIVST delivery but suggest tailored communication strategies for increasing the uptake of HIVST among these populations in countries with similar healthcare systems [16]. Particularly, there is an urgent need to design tailored interventions that increase the use of HIVST among people living in unsupportive social networks and those unwilling or unable to disclose their sexual or gender identities. Many studies outside the UK have reported the effectiveness of secondary distribution on the uptake of HIVST [17–19, 31, 33, 34]. We argue that such interventions may not reach people who experience unsupportive social networks, and who may benefit most from easy access to HIVST.

There are three main limitations to our study. Firstly, as this study only sampled half of the participants in SELPHI’s qualitative dataset, our findings may not fully represent SELPHI participants but depict conceptual typologies. In addition, our sampling strategy may overemphasise participants in the sub-study of Asian, Black and Latin American men, despite our efforts in presenting diverse perspectives from GBMSM and trans women. Secondly, our innovative typology requires careful interpretation, as it is only applicable to populations who are able to join online trials like SELPHI or live in countries with similar HIV testing services. Particularly, perspectives from resilient testers were based on a small sample (*n* = 4) with divergent accounts. Resilient testers were rarely identified in previous SELPHI research [24–27] as the innovative nature of HIVST tends to attract people who are either unsatisfied with HTS or living in social networks that facilitate information dissemination on HIV testing. To better understand this group, future research should explore the lived experiences of, and effective messages in promoting HIVST among, people with low unmet needs and unsupportive networks. Lastly, participants were subjected to recall bias due to the time between testing and interviews. As the majority of interviews were conducted before the COVID-19 pandemic, our findings cannot fully reflect users’ perspectives in the post-COVID era in which people may have more experience of self-testing generally.

## Conclusions

This study underscores the contextual differences in the HIVST experiences among GBMSM and trans women in England and Wales. Our proposed typology may facilitate the development of interventions tailored to the unmet needs of specific sub-groups of GBMSM and trans women. When planning HIVST programmes, policymakers should carefully consider individuals’ unmet testing needs and perceived levels of social support, particularly those who lack social support and struggle with utilising standard HIV testing and care services.

## Data Availability

Anonymised data are available upon reasonable request.

## Abbreviations

ABLA: Asian, Black and Latin American
GBMSM: Gay, Bisexual and other Men who have Sex with Men
HIVST: HIV Self-Testing
HTS: HIV Testing Services
RCT: Randomised Controlled Trial
SELPHI: HIV Self-testing Public Health Intervention
